# Dopaminergic Modulation of Striatal Inhibitory Transmission and Long-Term Plasticity

**DOI:** 10.1155/2015/789502

**Published:** 2015-07-29

**Authors:** Elizabeth Nieto Mendoza, Elizabeth Hernández Echeagaray

**Affiliations:** Unidad de Biomedicina, FES-I, Universidad Nacional Autónoma de México, Avenida de los Barrios No. 1, Los Reyes Iztacala, 54090 Tlalnepantla, MEX, Mexico

## Abstract

Dopamine (DA) modulates glutamatergic synaptic transmission and its plasticity in the striatum; however it is not well known how DA modulates long-term plasticity of striatal GABAergic inhibitory synapses. This work focused on the analysis of both dopaminergic modulation of inhibitory synapses and the synaptic plasticity established between GABAergic afferents to medium spiny neurons (MSNs). Our results showed that low and high DA concentrations mainly reduced the amplitude of inhibitory synaptic response; however detailed analysis of the D1 and D2 participation in this modulation displayed a wide variability in synaptic response. Analyzing DA participation in striatal GABAergic plasticity we observed that high frequency stimulation (HFS) of GABAergic interneurons in the presence of DA at a low concentration (200 nM) favored the expression of inhibitory striatal LTD, whereas higher concentration of DA (20 *μ*M) primarily induced LTP. Interestingly, the plasticity induced in an animal model of striatal degeneration mimicked that induced in the presence of DA at a high concentration, which was not abolished with D2 antagonist but was prevented by PKA blocker.

## 1. Introduction

Dopamine (DA) is involved in different functions of the nervous system like cognition, reward mechanisms, motor functions, learning, and memory. In the striatum, DA modulates synaptic transmission and synaptic plasticity through the activation of its DA receptors [1–4]. The modulation of DA depends on receptor subtype stimulated in a particular synapse; for example, activation of D1-class dopamine receptors (D1) increases glutamatergic responses mediated by N-methyl-D-aspartate (NMDA) and non-NMDA receptors in the corticostriatal pathway, and the stimulation of D2-class dopamine receptors (D2) attenuates them [5, 6]. Additionally, D1 and D2 activation is involved in the generation of long-term depression (LTD) and D1 contributes to long-term potentiating (LTP) of excitatory synapses of corticostriatal pathway [7, 8].

Although the striatum receives a massive amount of glutamatergic influences from the cortex and thalamus, MSNs, the projection cells, and most of local circuits of interneurons use GABA as a neurotransmitter [9]. GABAergic synapses on MSNs are also modulated by DA; for instance, activation of postsynaptic D1 receptors reduces GABA currents in MSNs [10], whereas the presynaptic activation of D1 receptors of axon collaterals of other MSNs increases the IPSC amplitude on MSNs and presynaptic D2 receptors decrease it [11].

Stimulation of MSNs or GABAergic interneurons with low and high frequency stimulation protocol induces short-term depression (STD) in striatal GABAergic synapses as the main form of synaptic plasticity [12, 13]. This short-term synaptic plasticity is also modulated by DA; activation of D1 receptors increases STD, while D2 agonist decreases it [14]. Long-term plasticity can be produced at inhibitory synapses on MSNs [15]; however, it remains unclear how DA modulates this type of plasticity.

Changes in DA content in the striatum are related to motor impairments, procedural learning, and cognitive deficits in animal models of neurodegeneration; low content of DA is associated with the development of Parkinson's Disease (PD), while high concentration of DA is related to symptoms of early stages of Huntington's Disease (HD) [16–18]; perhaps changes in synaptic plasticity underlie some of the deficits observed in such pathologies. Altered corticostriatal synaptic plasticity has been described after modifying DA receptors or DA content [8, 19], but it is not known how DA modulates inhibitory long-term synaptic plasticity on MSNs in striatal degeneration, nor if DA concentration is determinant to produce an specific type of plasticity.

In this study, DA effects on GABAergic inhibitory synaptic connections on principal neurons as well as in its synaptic long-term plasticity were analyzed. In addition, inhibitory long-term synaptic plasticity of an animal model of striatal degeneration was compared with the plasticity obtained using different concentrations of DA in control conditions.

## 2. Materials and Methods

### 2.1. Animals and Slice Preparation

Male C57/BL6 mice (Harlan Laboratories Inc., Mexico) ~40 days old and kept at room temperature (RT, 25°C) under a 12 : 12 h light : dark cycle with free access to food and water were used. To evaluate plasticity of inhibitory synapses on MSN in an animal model of striatal degeneration, some mice were treated with the mitochondrial toxin 3-nitropropionic acid (3-NP) for 5 days (15 mg/kg, once a day) as previously described [20, 21]. To identify MSNs that exhibited plasticity BACD1-GFP mice were used in some experiments. Experimental procedures were performed in accordance with international guidelines of animal care and the National Committee on Animal Research Ethics for the Care of Laboratory Animals (NOM-062-ZOO-1999).

Mice were ether-anesthetized in an induction chamber and later decapitated. Brains were removed and immersed in cold (4°C) artificial cerebral spinal fluid (CSF) bubbled with a mixture of 95% O_2_ and 5% CO_2_, containing the following composition (in mM): 26 NaHCO_3_, 1.25 NaH_2_PO_4_, 130 NaCl, 3 KCl, 5 MgCl_2_, 10 glucose, and 1 CaCl_2_, and maintained at a pH 7.4. Sagital brain slices (300 *μ*m) containing striatum were cut using a vibratome (Pelco 102, 1000 Plus model). Before experimental recordings, the slices were incubated (1 hr, RT) in the following artificial cerebrospinal fluid (ACSF; in mM): 26 NaHCO_3_, 1.25 NaH_2_PO_4_, 130 NaCl, 3 KCl, 2 MgCl_2_, 10 glucose, and 2 CaCl_2_, bubbled with 95% O_2_ and 5% CO_2_.

### 2.2. Electrophysiological Recordings

Electrophysiology was performed in a recording chamber perfused (2 mL/min) with ACSF bubbled with 95% O_2_/5% CO_2_, and cells were visualized using a microscope (BX51WI, Olympus, Germany) with DIC illumination, an infrared filter coupled to a CCD camera, and water immersion objective magnification (Olympus X Lum PlanFl 20x/0.95 W, Japan). MSNs of the dorsolateral striatum were whole-cell recorded using pulled (3–6 MΩ, Sutter Instruments, Inc., Model P-97) borosilicate pipettes (1B150F-4, World Precision Instruments, Inc.) filled with (in mM) 72 KH_2_PO_4_, 36 KCl, 2 MgCl_2_·6H_2_O, 10 HEPES, 1.1 EGTA, 0.2 Na_2_ATP, 0.2 Na_3_GTP, and 5 QX-314 to block unclamped action currents and 0.5% biocytin for further reconstruction, pH 7.2, 275 mOsm/L. Recordings were amplified (Axopatch 200B, Axon Instrument, Molecular Devices, USA), digitized (Digidata 1320 A, Axon Instrument, Molecular Devices, USA), and captured (5 kHz) using the pClamp 9.1 software (Axon Instrument, Molecular Devices, USA). Series and input resistance were compensated and monitored by evoking a transmembranal current with a voltage command during the experiment. Cells with unstable access resistance or more than 30 MΩ were excluded from the analysis.

**Figure 1:**
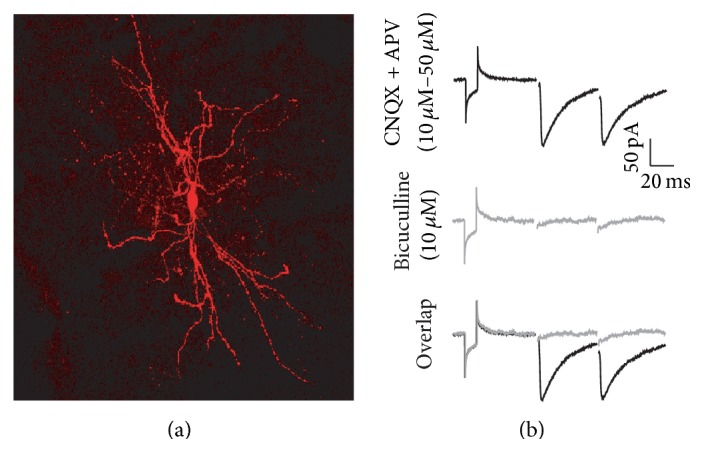
Characterization of IPSCs recordings in MSNs. (a) Reconstruction of a MSN filled with biocytin during electrophysiological recordings in voltage clamp mode and subsequently processed with avidin-Cy3. (b) Top: IPSCs of the MSNs in the presence of CNQX (10 *μ*M) and APV (50 *μ*M). Middle: Bicuculline (10 *μ*M) abolished the IPSCs of MSNs. Bottom: overlap of the recordings. *H*_*V*_ = −70 mV.

Inhibitory synaptic currents were evoked via a tungsten stimulating electrode (12 *μ*m tip; FHC, 0.1 Hz) positioned inside the striatum (100 *μ*m from the recording pipette) and connected to an isolation unit (DS2AK, Digimiter Ltd., Hertfordshire, UK). All recordings were conducted in MSNs (Figure 1(a), *H*_*V*_ = −70 mV) in the presence of glutamatergic antagonists (CNQX 10 *μ*m and APV 50 *μ*m) to isolate the GABAergic component, which was abolished in the presence of Bicuculline (10 *μ*M; Figure 1(b)). To induce striatal plasticity, a high frequency stimulation (HSF) protocol was used (3 trains, 3 seconds, of 100 Hz with an interval of 10 seconds between each train), and the cells were recorded for at least 40 min after the HFS.

### 2.3. Image Acquisition

Fluorescent images were obtained with a Hamamatsu (Orca C4742-95) camera coupled to the Olympus image acquisition system Cell M (excitation light 450–490 nm through a dichroic filter; emission light 502–538). Some slices were fixed and covered with Vectashield (Vector Laboratories, Burlingame, CA) for reconstruction. Visualization was made through a confocal fluorescence microscopy (Olympus Fv-1000) and acquired with the OLYMPUS FLUO VIEW 3.1 software.

### 2.4. Statistical Analysis

Data were analyzed and plotted offline using Microcal Origin 7 (Microcal Origin Lab Corporation, Northampton, MA, USA) and the statistical software Sigma Plot (Systat Software, Inc., San José, CA) with a parametric test or a nonparametric test if the data did not display a normal distribution. Data are expressed as mean ± SEM and significance was set at *p* < 0.05. The final figures were edited using Adobe Illustrator 10 or Adobe Creative Suite 5 (Adobe Systems, Inc., San José, CA).

## 3. Results

### 3.1. Dopaminergic Modulation of GABAergic Synaptic Transmission

MSNs of the striatum have two types of GABAergic synapses, those from axon collaterals of other MSNs and those from GABAergic interneurons. We performed intrastriatal stimulation; then, most of the GABAergic synaptic response was due to the stimulation of GABAergic interneurons [11, 14, 22, 23]. To determine the role of DA on these synapses, the effects of DA, DA agonists and antagonists on the GABAergic Inhibitory Postsynaptic Currents (IPSCs) were analyzed.

### 3.2. DA Modulation of Striatal GABAergic Transmission

**Figure 2:**
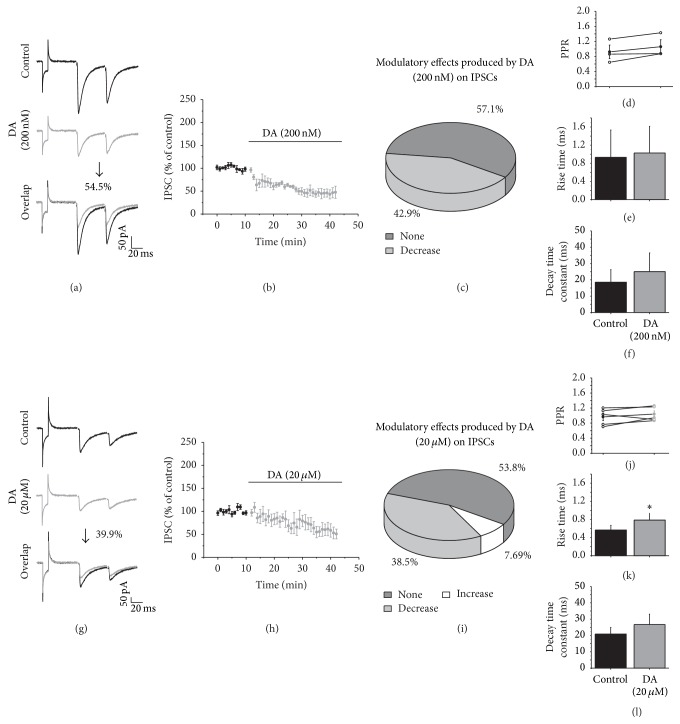
DA modulation of striatal GABAergic transmission. (a and g) show IPSCs traces in the control (top) and in the presence of DA ((a) 200 nM or (g) 20 *μ*M in the middle) and an overlap of the recordings (bottom). (b and h) illustrate the time course of the DA effects. Data are presented as percentage of change compared with the control in all graphs. (c and i) Pie charts illustrate the distribution of the modulatory effects of DA (200 nM and 20 *μ*M, resp.) on the IPSC amplitude. (d and i) display the PPR comparison of the IPSCs in the control and in the presence of DA. (e and k) The rise time and (f and l) the decay time constants of the IPSCs in the control and in the presence of DA. In this figure and the rest *H*_*V*_ = −70 mV, and recordings were in presence of CNQX (10 *μ*M) and APV (50 *μ*M).

Several studies have shown that DA modulatory effects on GABAergic transmission depend on the activation of different DA receptor subtypes [24, 25]. Then, to evaluate modulatory effects of DA on striatal GABAergic transmission, we studied the effect of DA treatment on MSNs using two different concentrations of DA (200 nM and 20 *μ*M) previously reported to have differential effects on modulation of GABAergic transmission (Li et al., 2012). Low DA concentration (200 nM) decreased IPSC amplitude compared with the control in 42.9% (*n* = 3) of the recorded cells (*t*_2_ = 5.968, *p* = 0.027; two-tailed paired *t*-test; Figures 2(a)–2(c)). In these cells, the PPR did not exhibit a significant change (Figure 2(d)), which would suggest a postsynaptic modulatory effect; however the kinetics current did not change in the presence of low DA concentration (Figures 2(e) and 2(f)) which suggests a presynaptic modulatory effect. The remaining recorded cells (*n* = 4, 57.1%) did not exhibit any amplitude change in the presence of low DA concentration (Figure 2(c)), suggesting that DA in low concentration did not modulate all GABAergic transmission on recorded MSNs.

Thirteen MSNs were evaluated with higher concentration of DA (20 *μ*M), and 53.8% (*n* = 7) of the recorded cells did not exhibit any modulation (Figure 2(i)), but 38.5% (*n* = 5) of the recorded cells did decrease the IPSC amplitude in its presence. Figures 2(g) and 2(h) illustrate that the IPSC amplitude was reduced by 39.9% from control amplitude (*t*_4_ = 3.150, *p* = 0.0345; two-tailed paired *t*-test; Figure 2(g)). The PPR did not exhibit significant changes (PPR: *t*_4_ = −1.171, *p* = 0.307; Figure 2(j)); in addition, the rise time significantly increased by 28% compared with control (rise time: *t*_4_ = 3.393, *p* = 0.0275; two-tailed paired *t*-test; Figure 2(k)), and the decay time constant remained the same (decay time: *t*_2_ = −1.928, *p* = 0.12; Figure 2(l)). This data suggests that 20 *μ*M of DA modulates GABAergic transmission through a postsynaptic mechanism. Only one cell (7.7%) exhibited an amplitude increase after the application of DA at the high concentration (data not shown).

### 3.3. D1 and D2 Receptor Activation Modulates Striatal GABAergic Transmission

In order to identify the DA receptor responsible for specific effects of GABAergic transmission modulation, it was evaluated in the presence of D1 or D2 agonist and antagonist.

**Figure 3:**
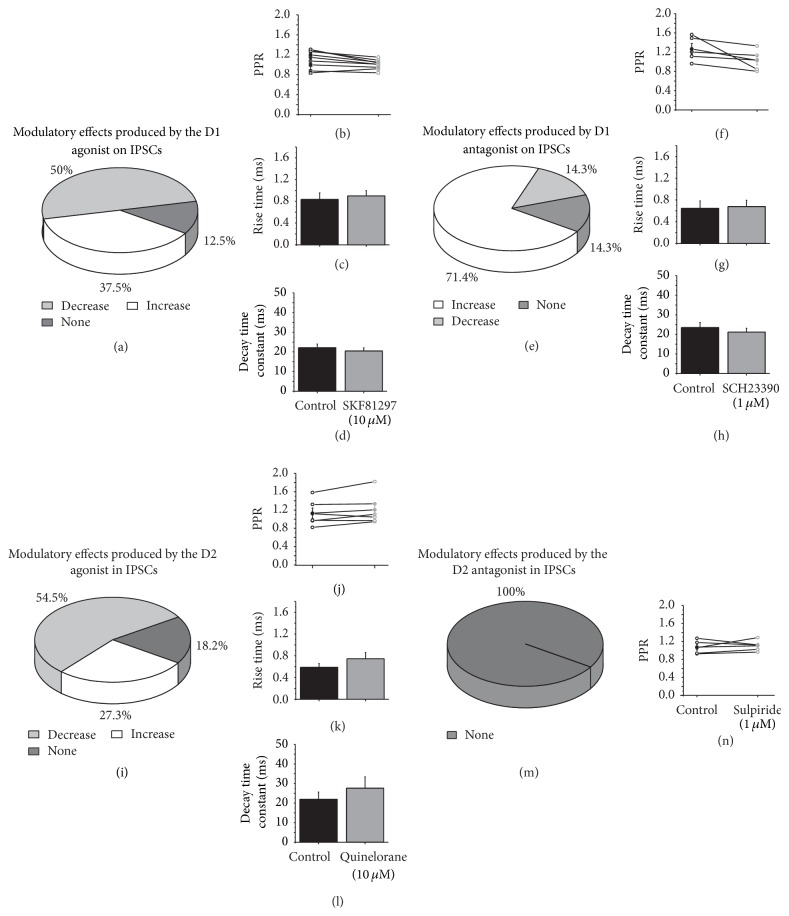
D1 and D2 receptors modulate striatal GABAergic transmission. (a, e, i, and m) are pie charts to illustrate the distribution of the modulatory effects of the D1 agonist, D1 antagonist, D2 agonist, and D2 antagonist, respectively, on the IPSC amplitude. (b, f, j, and n) display the PPR comparison of IPSCs in the control and after the addition of the DA reagent. (c, g, and k) The rise time and (d, h, and l) the decay time constants of the IPSCs in the control and in the presence of SKF81297 (10 *μ*M), SCH23390 (1 *μ*M), Quinelorane (10 *μ*M), and sulpiride (1 *μ*M).

In the presence of the D1 agonist (SKF81297 10 *μ*M), 50% (*n* = 4) of the recorded cells exhibited a decrease in the IPSC amplitude, 37.5% (*n* = 3) exhibited an increase, and 12.5% (*n* = 1) of the cells exhibit no change in the IPSC amplitude (Figure 3(a)). In the cells that exhibited an amplitude reduction, the amplitude was reduced by 27% compared with the control, and this difference was statistically significant (control 100.519 ± 0.880 versus SKF 73.694 ± 4.499, *t*_3_ = 5851, *p* = 0.001; two-tailed paired *t*-test). In the cells that exhibited an amplitude increase, the amplitude increased by 74% compared with the control (control 100.248 ± 0.599 versus SKF 174.361 ± 6.546, *t*_2_ = 2791, *p* = 0.049; two-tailed paired *t*-test).

Paired-pulse analysis and measurement of the rise time and the decay time of the IPSC were analyzed to determine the pre- or postsynaptic nature of the modulatory effect. For the recordings in which the amplitude of the IPSC increased, the paired-pulse ratio (PPR) did not change (PPR: *t*_2_ = 0.301, *p* = 0.778; two-tailed paired *t*-test; Figure 3(b)), nor did the time constants (rise time: *t*_2_ = −0246, *p* = 0.818; two-tailed paired *t*-test; Figure 3(c); decay time: *t*_2_ = 1677, *p* = 0.169; two-tailed paired *t*-test; Figure 3(d)). For those recordings in which the IPSC exhibited a decrease in the presence of the D1 agonist, the decrease in the PPR was not significantly different (*n* = 4, PPR: *t*_3_ = 2324, *p* = 0.059), nor were the changes in the kinetics (rise time: *t*_3_ = −0287, *p* = 0.784; two-tailed paired *t*-test; decay time: *t*_3_ = −0340, *p* = 0.746; two-tailed paired *t*-test). To construct the kinetics bars presented in Figures 3(c) and 3(d), all of the data from the cells were combined because they did not exhibit statistical changes in their kinetics. PPR exhibited no change in the presence of the D1 agonist, which may indicate a postsynaptic modulatory mechanism of the SKF81297; however the kinetics of the currents did not change in the presence of the D1 agonist which indicates a presynaptic mechanism for this modulation.

We next went to evaluate if endogenous DA affected IPSC amplitude by acting on D1 receptors; then D1 receptors were blocked. In the presence of the D1 antagonist, SCH23390 (1 *μ*M), the IPSC amplitude increased by 54% compared with the control (control 101.465 ± 0.816 versus SCH 155.553 ± 15.191; *t*_4_ = 3555, *p* = 0.007; two-tailed paired *t*-test) in 71.4% (*n* = 5) of the recorded cells (Figure 3(e)). The analysis of the PPR (Figure 3(f)) and time constants (Figures 3(g) and 3(h)) revealed that there were no significant changes. 14.3% (*n* = 1) of the cells exhibited a decrease in their IPSC amplitude in the presence of SCH23390, and 14.3% (*n* = 1) of the cells exhibited no change (data not shown). These data illustrated that endogenous DA reduced the IPSC amplitude in the majority of the recorded MSNs.

To evaluate if DA effects on IPSC amplitude were mediated by the activation of D2 receptors, 11 MSNs were evaluated in the presence of the D2 agonist (Quinelorane, 10 *μ*M). In this situation, the IPSC amplitude decreased in 54.5% (*n* = 6), increased in 27.3% (*n* = 3), and produced no effect in 18.2% (*n* = 2) of the recorded cells (Figure 3(i)). In the cells with an IPSC amplitude decrease, the amplitude was reduced by 41% compared with control and the modulation was statistically significant (control 100.11 ± 0.26 versus Quinelorane 58.69 ± 3.046, *t*_5_ = 13.246, *p* = 0.00004; two-tailed paired *t*-test; Figure 3(i)). These results indicated that DA through the activation of D2 receptors reduced GABAergic transmission. The PPR did not change suggesting a postsynaptic mechanism (Figure 3(j)), nor however the IPSC kinetics did, suggesting a presynaptic mediated mechanism (Figures 3(k) and 3(l)).

To evaluate if endogenous DA affected IPSC amplitude through D2 activated mechanisms, the D2 antagonist sulpiride (1 *μ*M) was studied. Sulpiride produced no change in the IPSC amplitude, PPR, or current kinetics (*n* = 5, Figures 3(m) and 3(n)). These experiments illustrated that endogenous DA do not modulate IPSC amplitude by D2 activation.

### 3.4. GABAergic Synaptic Plasticity

**Figure 4:**
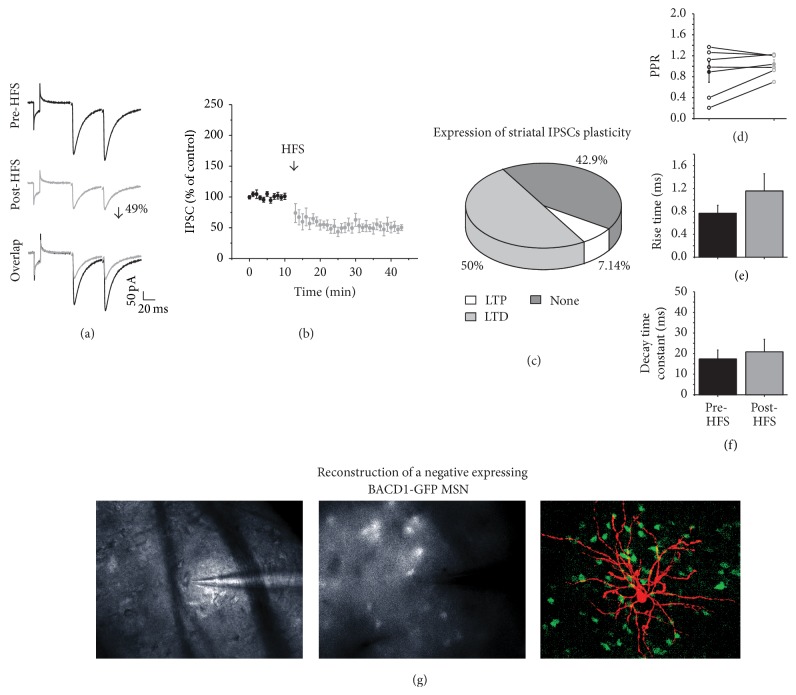
GABAergic synaptic plasticity. (a) shows representative traces of IPSC before and after the HFS (3 trains, of 100 Hz, for 3 s, with 10 s of interval). (b) Time course of the IPSC amplitudes before and after HFS. The data are normalized and presented as the percentage of change compared with the control in this figure and the rest of figures. (c) Distribution, in percentages, of the type of plasticity generated by HFS, 50% developed LTD, 7.14% developed LTP, and 42.9 did not develop plasticity. (d) PPR comparison of the IPSCs before and after HFS did not change. (e) Rise time and (f) decay time constants before and after stimulation. (g) Reconstruction of a MSN that exhibited LTD but was not positive to D1-GFP. In the left 10x magnification of the cell, in the middle 60x augmentation, note that no fluorescence is observed in the tip of the electrode. In the right, the cell was filled with biocytin during the electrophysiological recording and later processed with avidin-Cy3, to visualize it. Note that there is no overlap between GFP and the Cy3 of the MSN.

To analyze the synaptic plasticity of the IPSCs in MSNs, HFS was given to 14 MSNs cells (see Section 2).  Figure 4(a) presents the IPSC amplitude before (top) and after HFS (middle). HFS significantly decreased the IPSC amplitude by 49% compared with the amplitude before the train (*t*_6_ = 5919, *p* < 0.001), in 50% (*n* = 7) of the recorded cells; this reduction persisted for more than 30 minutes; then, the current amplitude decrease was considered to be long-term depression (LTD; Figure 4(b)). The PPR before and after HFS did not change (*n* = 7; Figure 4(d)) nor did the time constants (Figures 4(e) and 4(f)). 42.9% (*n* = 6) of the recorded cells showed no change in the current amplitude after HFS, and only 7.14% (*n* = 1) of recorded cells exhibited an amplitude increase (Figure 4(c)).

To evaluate if all types of MSN developed inhibitory long-term plasticity, some experiments were performed on slices coming from BACD1-GFP mice. 13 cells were recorded, 4 cells did not exhibit any plasticity, and both BACD1-GFP-positive (*n* = 3) and BACD1-GFP-negative (*n* = 3) MSNs developed LTD after HFS; no one of the evaluated BACD1-GFP-positive cells developed LTP; however 3 BACD1-GFP-*negative* cells developed LTP.  Figure 4(g) illustrates the reconstruction of a D1-*GFP-negative expressing* neuron that displayed GABAergic LTD; this neuron was considered as D2 expressing MSN.

From these results we conclude that MSNs expressing D1 and D2 developed LTD as the prevalent form of inhibitory long-term plasticity, and only neurons expressing D2 receptors displayed LTP.

### 3.5. GABAergic Synaptic Plasticity in Presence of DA

**Figure 5:**
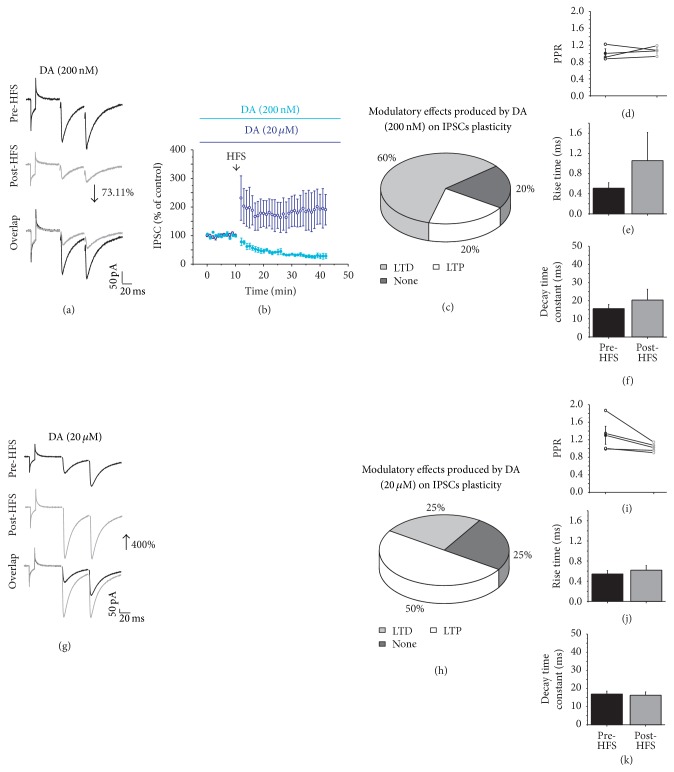
DA modulates GABAergic synaptic plasticity of MSNs. (a and g) are representative IPSC traces in the presence of DA (200 nM or 20 *μ*M, resp.) before (top) and after HFS (middle) and an overlap of the recordings (bottom). (b) Time course of the IPSC amplitude before and after HFS in the presence of DA 200 nM (light blue) and 20 *μ*M (dark blue). (c and h) show the distribution, in percentages, of the types of plasticity that were generated in the presence of DA (200 nM or 20 *μ*M, resp.). (d and i) are the PPR comparisons of the IPSCs before and after HFS in the presence of DA (200 nM or 20 *μ*M). (e and j) are the rise time, while (f and k) are the decay time before and after HFS in the presence of DA (200 nM or 20 *μ*M).

Once we showed that GABAergic synapses exhibit LTD as the main form of synaptic plasticity, we investigated whether DA modulate this form of plasticity. The role of DA in modulating GABAergic plasticity was also evaluated using two concentrations (200 nM and 20 *μ*M). In the presence of a low concentration of DA (200 nM), HFS induced LTD (Figure 5(a)) in 60% (*n* = 3) of the recorded cells, LTP was produced in only 20% (*n* = 1) of the recorded cells (Figure 5(c)), and 20% (*n* = 1) did not displayed any plasticity. In the cells in which LTD was observed, the amplitude was reduced by 73.11% compared with the current amplitude before HFS (*t*_2_ = 10.255, *p* = 0.00938; Figures 5(a) and 5(b)). DA (200 nM) did not generate changes in the PPR nor in the current kinetics (Figures 5(d)–5(f)). High concentration of DA (20 *μ*M) induced a significant increase in the IPSC amplitude after HFS (*t*_3_ = 1.826, *p* = 0.125, Figures 5(b) and 5(g)) in 50% (*n* = 4) of the evaluated cells (Figure 5(h)). The PPR and current kinetics did not change (Figures 5(i)–5(k)).

### 3.6. D1 Receptors Modulation of Striatal GABAergic Synaptic Plasticity

**Figure 6:**
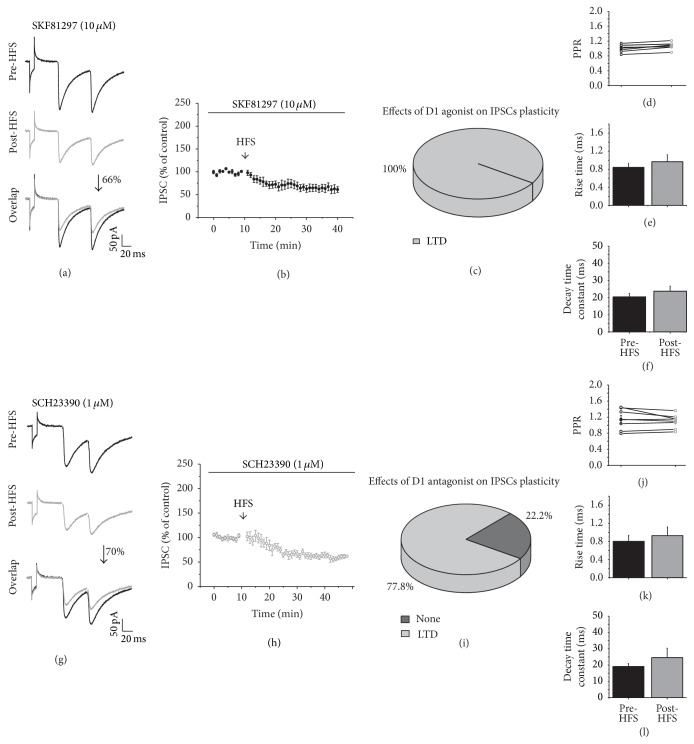
D1 modulation of GABAergic synaptic plasticity. (a and g) show representative IPSC traces before HFS (top) and after HFS (middle) and an overlap of the recordings (bottom), all in the presence of the D1 agonist SKF81297 (10 *μ*M) and the D1 antagonist SCH23390 (1 *μ*M), respectively. (b and h) illustrate the time course of the effects of HFS on the IPSC amplitude in the presence of SKF81297 and SCH23390, respectively. (c and i) display the distribution in percentages of the types of plasticity that were generated in the presence of the D1 agonist or antagonist. (d and j) are the PPR comparison of the IPSCs before and after HFS in presence of SKF81297 and SCH23390, respectively. (e and k) are the rise time, while (f and l) are the decay time in the presence of SKF81297 and SCH23390, respectively, before and after HFS.

To better analyze DA effects of plasticity we used specific agonist and antagonist of dopamine receptors. In the presence of the D1 agonist SKF81297 (10 *μ*M), HFS induced LTD in all of the recorded cells (*n* = 7, *t*_6_ = 77, *p* < 0.001; two-tailed paired *t*-test; Figures 6(a)–6(c)). The PPR (Figure 6(d)), rise time, and decay time did not change (Figures 6(e) and 6(f)) after HFS, indicating that D1 agonist activation modulates the LTD expression on striatal inhibitory plasticity through presynaptic mechanisms.

In the presence of the D1 antagonist, SCH23390 (1 *μ*M), HFS induced LTD in 77. 8% (*n* = 7) of the recorded cells (*t*_6_ = 10.042; *p* < 0.001; two-tailed paired *t*-test, Figures 6(g)–6(i)). The PPR (Figure 6(j)) and the time constants of the currents (Figures 6(k) and 6(l)) did not change. 22.2% (*n* = 2) of the recorded cells in the presence of the D1 antagonist did not develop any plasticity (Figure 6(i)). If D1 receptor activation was responsible for generating LTD, the block of the D1 receptors would eliminate LTD; however, 77.8% of the cells remained producing LTD (Figure 6(i)); then, receptors other than D1 should favor LTD on inhibitory synapses.

### 3.7. D2 Receptor Modulation of Striatal GABAergic Synaptic Plasticity

**Figure 7:**
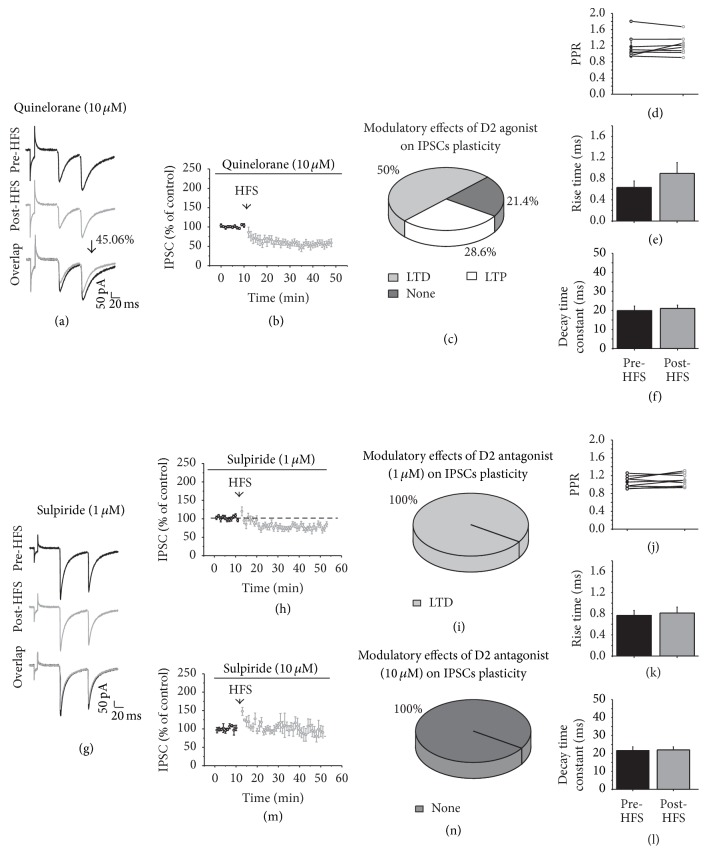
D2 modulation of GABAergic synaptic plasticity. (a and g) show representative IPSC traces before (top) and after HFS (middle) in the presence of the D2 agonist (Quinelorane, 10 *μ*M) and the D2 antagonist (sulpiride, 1 *μ*M), respectively, and an overlap of the recordings (bottom). (b, h, and m) are the time course of the IPSC amplitude before and after HFS in the presence of Quinelorane or sulpiride. (c and i) illustrate the distribution, in percentages, of the types of plasticity that were generated in the presence of the D2 agents. (d and j) are the PPR comparison of the IPSCs before and after HFS in presence of Quinelorane or sulpiride. (e and k) are the rise time and (f and l) are the decay time in the presence of Quinelorane or sulpiride before and after HFS. (n) HFS in the presence of sulpiride blocks the generation of LTD.

We next went to evaluate D2 receptors role in striatal plasticity of inhibitory synapses on MSN. In the presence of the D2 agonist Quinelorane (10 *μ*M), HFS generated LTD in 50% (*n* = 7) of the recorded cells and LTP in 28.6% (*n* = 4) of them. In those cells where LTD was generated, the amplitude of IPSC was reduced by 45.06% compared with the amplitude before HFS (Pre-HFS = 100.895 ± 0.687 versus Post-HFS = 55.833 ± 7.316; *t*_6_ = 5.838, *p* = 0.001; two-tailed paired *t*-test, Figures 7(a) and 7(b)). The LTD did not produce changes in the PPR or in the current kinetics (Figures 7(d)–7(f)). In those cells that exhibited LTP in the presence of Quinelorane (Figure 7(c)), the increase in the IPSC amplitude was statistically significant (*t*_3_ = 5685, *p* = 0.002). The PPR significantly decreased (*t*_3_ = 2.398, *p* = 0.0373) without any change in the current kinetics, indicative of presynaptic mechanisms in D2 modulation of LTP (data not shown). The rest of the recorded cells (21.4%, *n* = 3) demonstrated no change after HFS (Figure 7(c)). Data obtained in this section suggested that D2 receptor participates in the induction of LTD but also in LTP of GABAergic plasticity because the percentage of cells that exhibited LTP increased from 7.14 in control conditions to 28.6%.

**Figure 8:**
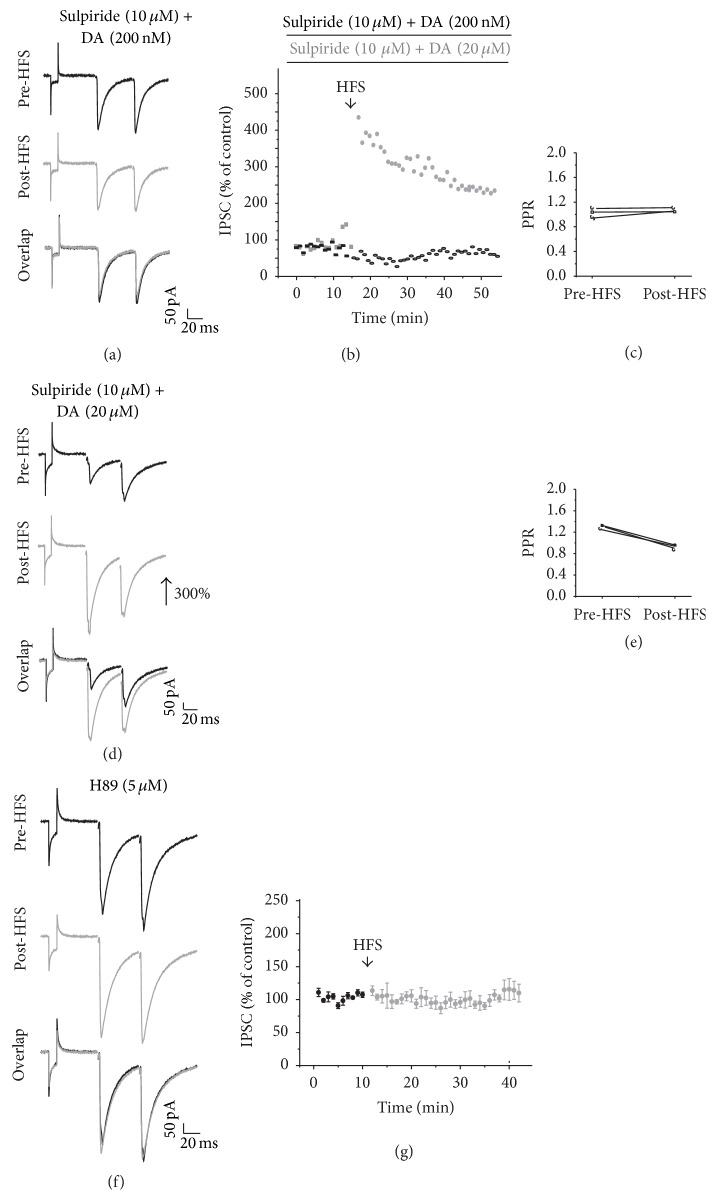
Sulpiride and H89 on DA triggered plasticity. (a and d) are representative IPSC traces in the presence of DA + sulpiride before (top) and after HFS (middle) and the overlap of the recordings (bottom). (b) Time course of the IPSC amplitude of two experiments before and after HFS in the presence of DA (200 nM) + sulpiride (10 *μ*M) and DA (20 *μ*M) + sulpiride (10 *μ*M). Note that sulpiride prevented the LTD induced by DA 200 nM but did not prevent the LTP induced by HFS in the presence of DA (20 *μ*M). (c and e) are the PPR comparison of the IPSCs before and after HFS in presence of DA (200 nM or 20 *μ*M, resp.) + sulpiride (10 *μ*M). (f) illustrates representative IPSC traces in the presence of PKA inhibitor H89. (g) Time course of the IPSC amplitude in the presence of the PKA blocker. Note that H89 blocks striatal plasticity induction.

In the presence of the antagonist D2 sulpiride (1 *μ*M), HFS generated a modest but statistically significant LTD in all of the recorded cells (*n* = 8, Pre-HFS = 102.107 ± 1.36 versus Post-HFS = 74.28 ± 6.25; *t*_7_ = 4.933, *p* = 0.00169; two-tailed *t*-test; Figures 8(g), 8(h), and 8(i)); however the LTD generated in the presence of sulpiride was slightly compared with that observed with Quinelorane (IPSC reduced by 45.06% in the presence of Quinelorane and 27.8% in the presence of sulpiride), suggesting that sulpiride reduced part of the LTD generated by D2 stimulation. The PPR did not change (*n* = 8; Figure 7(j)) nor did the time constants (*n* = 8; Figures 7(k) and 7(l)). Higher concentration of sulpiride (10 *μ*M) was needed to fully prevent the LTD induction after HFS (*n* = 5; Pre-HFS = 100.47 0.21 versus Post-HFS = 118.82 ± 15.70, *t*_4_ = −1.182, *p* = 0.303; two-tailed *t*-test; Figures 7(m) and 7(n)). This data demonstrated that the block of D2 receptors prevented LTD induction in inhibitory synapses on MSN.

Once knowing that the D2 antagonist abolished the development of LTD after HFS protocol, we evaluated the effect of HFS in the presence of both DA 200 nM and the D2 antagonist sulpiride (10 *μ*M). Under this condition, sulpiride prevented the LTD generated by HFS in presence of DA (200 nM) (*n* = 3; Figures 8(a) and 8(b)), without changes in the PPR (Figure 8(c)). Conversely, in the presence of DA (20 *μ*M), sulpiride did not affect the LTP generated by HFS (*n* = 3; Figures 8(b) and 8(d)) and no changes were obtained in the PPR. Interestingly, PKA inhibitor H89 inside the recording pipette prevented the induction of any plasticity (*n* = 5, Pre-HFS = 103.33 ± 1.66; Post-HFS = 103.86 ± 8.66; *t*_4_ = −0.0617, *p* = 0.954; two-tailed *t*-test; Figures 8(f) and 8(g)).

### 3.8. GABAergic Synaptic Plasticity in Striatal Degeneration: Role of DA

**Figure 9:**
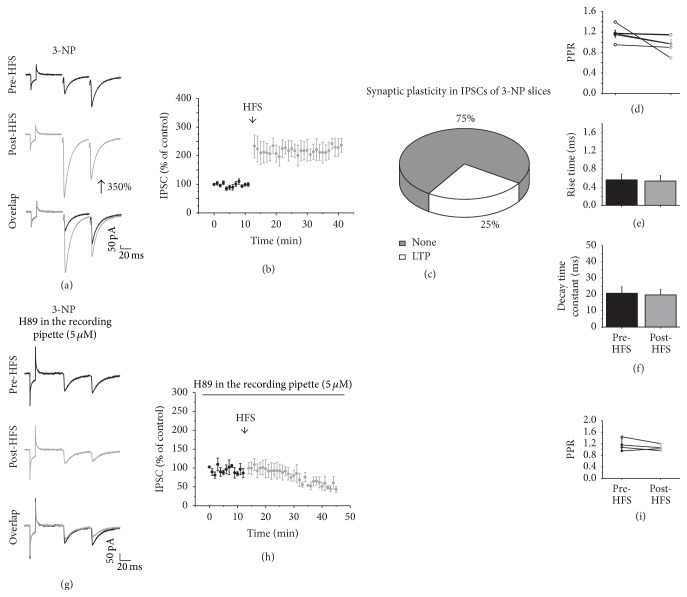
Synaptic plasticity in the striatal degeneration. (a) shows representative traces of IPSC before (top) and after HFS (middle) and an overlap of the traces (bottom). Note that LTP is produced after HFS. (b) illustrates the time course of the IPSC amplitude before and after HFS. (c) displays the percentage of cells that exhibited LTP after HFS in 3-NP-treated slices. (d) shows the PPR comparison of the IPSCs before and after HFS. (e) The rise time and (f) decay time constants before and after HFS. (g) shows representative IPSCs traces before (top) and after HFS (middle) in the presence of H89 (5 *μ*M) and overlap of the traces (bottom). (h) Time course of the IPSC amplitude before and after HFS in the presence of H89 (5 *μ*M). Note that the block of PKA prevented the generation of LTP in 3-NP slices. (i) is the PPR comparison of the IPSCs before and after HFS in the presence of H89.

High levels of DA have been observed in early stages of striatal degeneration such as HD, and then we wondered if GABAergic plasticity in such conditions could be similar to that observed under high DA concentration. Therefore, some experiments were designed to analyze the type of plasticity triggered in the IPSCs during the striatal degeneration induced by the systemic administration of 3-NP (15 mg/kg, i.p., 5 days). Of the 20 recorded cells from 3-NP-treated mice slices, only 5 of the recorded cells (25%) exhibited synaptic plasticity (Figure 9(c)). Interestingly, the type of plasticity generated was only LTP (Pre-HFS = 97.45 ± 1.73 versus Post-HFS = 220.45 ± 26.32; *t*_4_ = −4.565; *p* = 0.0103, Figures 9(a) and 9(b)). This result was similar to that obtained in the presence of DA (20 *μ*M). No changes were observed in the PPR (Figure 9(d)) or in the current kinetics before and after HFS (Figures 8(e) and 8(f)). In the presence of the D1 antagonist, LTP induction was prevented in cells from 3-NP-treated mice (Pre-HFS = 98.87 ± 1.26 versus Post-HSF = 105.19 ± 8.62; *t*_8_ = −0.725, *p* = 0.489; two-tailed *t*-test, data not shown). Furthermore, as the block of PKA signaling pathway prevented the LTP induced in the presence of DA in high concentration (20 *μ*M, Figure 8(g)), we performed some experiments with the PKA inhibitor H89 (5 *μ*M) inside of the recording pipette in slices from 3-NP-treated mice. In this situation, the LTP induction after HFS was prevented (Figures 9(g) and 9(h)) and LTD development was favored (Pre-HFS = 98.16 ± 2.95 versus Post-HFS = 55.22 ± 9.54; *t*_2_ = 6.52; *p* = 0.02; two-tailed *t*-test) without changes in the PPR (Figure 9(i)). This result supports the idea that LTP of inhibitory synapses on MSNs is triggered by PKA signaling pathway in striatal degeneration and its postsynaptic inhibition promotes the development of LTD.

## 4. Discussion

The present study describes that DA modulation of GABAergic synapses on MSNs and the type of plasticity developed depend on the DA concentration and the activation of D1- or D2-class receptors. Also DA effect on striatal plasticity was altered in striatal degeneration.

### 4.1. DA Modulation of GABAergic Synapses on MSNs

DA at a low and high concentration decreased the IPSC amplitude compared with the control conditions in most of the recorded cells. However when specific D1 and D2 agents were evaluated modulation on GABAergic synapses depended on DA receptors activation or inhibition. The D1 agonist produced decrease and increase in the IPSC amplitude of the MSNs recorded. The D1 family receptors include the D_1_ and D_5_ receptors, which are differentially located in striatal neurons, and exhibited different affinities for the agonist [26] and different dependencies on the G protein/adenylyl cyclase signaling pathway [27]. Additionally, there are also diverse responses due to the activation of pre- or postsynaptic mechanisms. In recordings from dissociated MSNs, SKF81297 (1–10 *μ*M) reduces the postsynaptic GABA ligand-gated-currents [10, 28]. On the contrary, the presynaptic D_1_ receptor activation of axonal collaterals from MSNs stimulates GABAergic synapses on other MSNs [29]. Furthermore, D_5_ receptors are expressed in the terminals of GABAergic interneurons [30], because most of the cells recruited with intrastriatal stimulation are the GABAergic interneurons [23]; changes in the IPSC amplitude in the presence of the D1 agonist may be due to the activation of the presynaptic D_5_ receptors on GABAergic interneurons.

Blocking the D1 receptors mainly produced an increase in the GABAergic currents, suggesting that when D1 receptors are blocked, the D2 activation by endogenous DA is unmasked. The agonist D2, Quinelorane 10 *μ*M, also produces decrease and increase of the IPSC amplitude. D2 receptors belong to a family that has several subtypes, such as D_2_, D_3_, and D_4_; the striatum mostly expresses D_2_ and D_3_ [26] and Quinelorane affects both of them and may produce different responses depending on receptor sensitivity and selectivity [31]. D2 receptors are located postsynaptically in the MSNs that express enkephalins, but they are also autoreceptors in the striatal dopaminergic terminals; low concentrations of DA activate them, decreasing the endogenous release of DA as a response [26]. Subsequently, in those cells in which the D2 agonist produced an IPSC amplitude increase, the effect may be postsynaptically mediated, whereas a decrease in the IPSC may be related to a presynaptic effect. Earlier study showed that the stimulation of presynaptic D2 receptors decreases GABAergic synaptic amplitude [29].

### 4.2. GABAergic Synaptic Plasticity and Its DA Modulation

HFS decreased the IPSC amplitude in 50% of the experiments; these results are consistent with those obtained by others using different stimulation protocols [32, 33].

DA concentration was crucial for determining the type of inhibitory plasticity triggered at the striatum. In the presence of DA, HFS generated both LTD and LTP; however, a low DA concentration (200 nM) produced mostly LTD, whereas higher concentration of DA (20 *μ*M) favored LTP in half of the experiments. DA at low concentrations activates high affinity receptors, whereas higher concentrations also activate low affinity receptors. D2 receptors possess higher affinity for DA and their stimulation facilitates LTD. Moreover, the administration of sulpiride 10 *μ*M prevented the LTD induced with HFS in the presence of DA at 200 nM, whereas the administration of sulpiride in the presence of DA at 20 *μ*M did not prevent the induction of LTP, but only H89 did, supporting the notion that D2 receptors play an important role in triggering striatal LTD in inhibitory synapses at the striatum, whereas D1 activation coupled to PKA signaling pathway is mainly involved in generating LTP in the GABAergic synapses. However, we cannot rule out that postsynaptic D2 receptors may mediate increases in the IPSCs after HFS in the presence of 20 *μ*M of DA because postsynaptic D2 in MSNs of the indirect pathway can stimulate intracellular calcium through the phospholipase C pathway [34]. This pathway enhances GABA_A_ currents by mobilizing intracellular Ca^2+^ [35].

### 4.3. D1 and D2 Modulation of Striatal GABAergic Synaptic Plasticity

To better understand DA receptors role in plasticity, specific DA agonist and antagonist were evaluated. In the presence of the D1 agonist, HFS produced LTD in all recorded cells. Despite this finding, HFS in the presence of the D1 antagonist still generated LTD in a large percentage of the cells (77.8%), demonstrating that the inhibition of D1 receptors did not prevent the development of striatal LTD in the majority of the inhibitory synapses.

HFS in the presence of the D2 agonist generated LTD in 50% of the cells and LTP in 28% of the cells. Nevertheless, in the presence of the D2 antagonist, all Inhibitory plasticity in MSNs was prevented. In fact, in D2 knockout mice LTD of corticostriatal excitatory plasticity is abolished, generating LTP instead [36]. Our results indicate that D2 receptors play an important role in triggering LTD of inhibitory striatal plasticity as well.

### 4.4. GABAergic Plasticity in Striatal Degeneration

Synaptic abnormalities in corticostriatal pathway have been described after acute 3-NP treatment [21, 37, 38], but there is no study of changes on inhibitory synapses on MSNs. Evaluation of synaptic plasticity of this connection on damaged MSNs indicated that 75% of the recorded cells did not exhibit any plasticity, while the other 25% of the cells exhibited only LTP. The LTP produced in slices of 3-NP-treated mice resembled the LTP triggered under high levels of DA in normal tissue (Figures 9(b) and 5(b), resp.). This LTP was affected by the D1 antagonist and the PKA inhibitor (Figures 9(g) and 9(h)) which in its presence generates LTD, suggesting that D1 receptors are involved in the plasticity observed in damaged striatal tissue. Striatal LTP in IPSCs may result from a dysregulation in DA release [39] produced by 3-NP, as observed in other animal models of striatal degeneration [19]. It appears that a sustained elevation of DA or an imbalance in its concentration causes selective degeneration of striatal GABAergic neurons and motor dysfunction [17, 18]. A reduction in D1 receptors has been documented in acute 3-NP administration [37]; if this were the case within* in vivo* subchronic treatment, an alternative mechanism to explain LTP in striatal IPSCs is that feed forward inhibition mechanism, mediated mainly by fast spiking (FS) interneurons [40] on MSNs expressing D1 receptors (direct pathway), was overactivated in damaged striatum; in fact we have shown a reduction in number of spines, and dendrites tick in our 3-NP model of degeneration [21] which may indicate that interneurons projecting on the perisomatic area would have more synaptic impact on MSNs than those arriving in distal areas; FS are the ones heavily innervating perisomatic area in MSNs [9]; then LTP exhibited in MSN expressing D1 receptors may be produced when FS are activated with field stimulation in control slices as well as in slices from damaged striatum; further experiments should test this hypothesis.

## 5. Conclusion

DA effect on striatal plasticity was different from its modulatory action in inhibitory synapses on MSNs. The variability on synaptic responses may be due in part to the set of GABAergic afferents to MSNs (mainly interneurons) stimulated that exhibit different DA receptors as well as DA receptors that are present in recorded MSNs.
