# Corrigendum to “Dopaminergic Modulation of Striatal Inhibitory Transmission and Long-Term Plasticity”

**DOI:** 10.1155/2017/3143428

**Published:** 2017-03-02

**Authors:** Elizabeth Nieto-Mendoza, Elizabeth Hernández-Echeagaray

**Affiliations:** Unidad de Biomedicina, FES-I, Universidad Nacional Autónoma de México, Avenida de los Barrios No. 1, Los Reyes Iztacala, 54090 Tlalnepantla, MEX, Mexico

In the article titled “Dopaminergic Modulation of Striatal Inhibitory Transmission and Long-Term Plasticity” [1], the names of the authors are corrected as above and there was an error in the Acknowledgments section, which should be corrected as follows.

This work was supported by CONACyT Grant no. 81062-2007 and DGAPA-PAPIIT Grant no. IN201307. Elizabeth Nieto Mendoza received a Ph.D. fellowship from CONACyT-México and a thesis fellowship from COMECyT. This work is a partial fulfillment of the requirements for her Ph.D. degree in Ciencias Biomédicas at Universidad Nacional Autónoma de México. The authors thank the Mutant Mouse Regional Resource Center U42OD010918, University of Missouri, for producing BACD1-GFP; C. Rivera and X. Ayala for breeding them in the animal facility of the IFC, UNAM; E. Mendoza and D. Tapia for technical support during the experimental procedures and for histological processing, respectively; and Dr. J. Bargas (IFC, UNAM) for his comments on this work.
